# Unpacking boredom factors of Chinese foreign language major students in translation classes: A sequential mixed methods study

**DOI:** 10.3389/fpsyg.2022.895223

**Published:** 2022-09-15

**Authors:** Tingyu Zhang

**Affiliations:** School of English and International Studies, Beijing Foreign Studies University, Beijing, China

**Keywords:** Chinese foreign language major students, boredom factors, emotion study, translation classroom, translator cultivation

## Abstract

Though largely ignored by educators and researchers, boredom, an aversive emotion, is the hurdle for many translation learners to be professional. It is all the more important to unpack the boredom factors of Chinese foreign language major students in translation classes, which will serve in promoting students’ engagement in L2 classes and translator cultivation. By using a sequential mixed approach, this study conducted a thematic analysis and built a structural equation model. Quantitative data were gleaned from 483 foreign major college students in China and qualitative data were selected from students (*N* = 15) of 7 universities through snowball sampling. Initially, the first-order confirmatory factor analysis (CFA) verified the adapted questionnaire and the second-order CFA finds each factor’s contribution. Then, the thematic analysis gives support to the empirical findings. Both quantitative and qualitative analyses revealed that task-based boredom is the most routinely experienced boredom. Moreover, two major sources of boredom in translation classes are found, including task-based boredom and teacher-related boredom. It is suggested that teachers should improve their classes and use more interesting and engaging materials and tasks with the proper level of difficulty. The finding can help educators to identify the major boredom in the translation class and help potential professional translators to grow. Future studies will also be facilitated by the present study to find approaches to tackle these boredom sources.

## Introduction

Emotion in second language acquisition (SLA) has been enjoying increasing scholarly attention over the years. The recognition of its various impacts on learners in SLA and other fields has been more intensified in that emotion is considered to determine learners’ achievement and navigate the whole learning process (Dewaele, 2019; Li et al., 2019; Dewaele and Li, 2020).

Emotions are classified into two major types. The first category is the positive emotion, namely love, hope, pride, enjoyment, excitement, etc. The other is negative emotion including but not limited to shame, guilt, and burnout. Traditionally, researchers have been heavily focusing on anxiety and burnout when studying emotions (Marcos-Llinás and Garau, 2009; Vaezi and Fallah, 2011). Gradually, scholars began to identify the partiality of this ever-present focus and shift their views to other underexplored emotions in EFL/ESL contexts (Wang et al., 2021). Boredom is one of them, with a growing number of studies on its measurements and effects. In the educational context, boredom frequently occurs such as to severely compromise the learner’s efficiency (Pawlak et al., 2020).

This accords with the experience of many Chinese English as a foreign language students who fail to cooperate with the teacher in the L2 classes, especially in the translation classroom. The situation is even worse for those who major in foreign languages. This may attribute to the intensive training in translation classes. Learners need rigorous attention to encounter ponderous pressure when doing the translation. Thereby, they easily become exhausted in translation class.

However, as there’s a paucity of literature on boredom in translation class, the exact factors of boredom in the translation classroom remain unknown, which enables this obstructive emotion to impact students continuously. Plus, teachers and researchers always undervalue boredom and confuse it with anxiety and laziness.

Yet, it is worth noting that the study of translation classrooms is of paramount significance because it can help foreign language major students to understand the source language and refine their language skills, contributing to the construction of large translator talent pools. As such, unraveling the factors of boredom in the translation classroom plays a vital role in providing students who major in foreign languages with instructions to improve the effectiveness of learning translation. On top of that, a related in-depth exploration can also assist teachers in recognizing the source to instigate boredom in the translation classroom.

Therefore, this study used a sequential mixed method with an adapted questionnaire to investigate foreign language major students in China in terms of their boredom factors in the translation classroom.

## Literature review

### Definition of boredom

Boredom refers to individuals’ psychologically or emotionally dreary situation, which always entails deleterious effects on motivation, engagement, and curiosity (Dewaele, 2019; Dewaele and Li, 2020). It is a melting pot of discontent, tiredness, anxiety, and anger, accounting for profuse dimensions (Weinerman and Kenner, 2016; Kruk and Zawodniak, 2018). As for its construct, it is multi-layered and always observed with learners’ non-engagement in class and blunt perception of time lapses (Derakhshan et al., 2021).

This feature of boredom prevents itself from being valued by scholars and poses challenges for researchers to give a solid definition because of various psychological, sociological, and educational factors. Nevertheless, the majority of the academic circle has reached a consensus that boredom is silent, unseen, underestimated but detrimental to students’ academic performance and attainment (Li et al., 2021).

### Types of boredom

Boredom can be classified into many types. Firstly, sorted by the degree of valence and arousal, there is indifferent boredom, calibrating boredom, searching boredom, reactant boredom, and apathetic boredom (Goetz et al., 2014). Secondly, based on different situations, classroom boredom and homework boredom can be distinguished (Macklem, 2015). Thirdly, regarding the relations among people, a line can be drawn between personal boredom and interpersonal boredom (Tempelaar and Niculescu, 2021). Personal boredom can be understood as the boredom within a person. Interpersonal boredom refers to the boredom brought by others. Finally, according to Putwain et al. (2018), grounded on the lengthen of boredom, there is trait boredom and state boredom. Trait boredom is subject-related or activity-related, always lasting for long period. In comparison, state boredom is short-term, always triggered by a certain context.

To sum up, boredom can be categorized from perspectives of stimulus, situations, human relations, and boredom length.

### Causes and measurements of the boredom

Many scholars have tried to develop models to explain the causes of academic boredom. Despite the diversities, theories can be recognized as two kinds: people-centered and environment-based.

For the first category, the focus is on causes around persons, such as psychological energy, ability, etc. There is the control-value theory, the mention theory, and the attentional theory. Specifically, the control-value theory is based on the premise that achievement emotions are triggered by the appraisals of control and values (Pekrun, 2006). This theory holds that learners will experience boredom frequently because of the activity itself or the unsatisfied outcome. Boredom is posited to be instigated when leaner consider the activity or learning material as irrelevant, unimportant, or invaluable (Li, 2021). Likewise, the mention theory believes boredom appears when students have inadequate energy to cope with overwhelmingly challenging tasks (Davies and Fortney, 2012). The attentional theory argues that boredom occurs when learners cannot control their attention and they are unable to focus on the lessons (Eastwood et al., 2012). These theories are people-oriented, implying that boredom comes from within people. Though they offered more scholarly insights into the source of boredom, external factors such as learning equipment, teaching method, and educators’ attitude are not sufficiently addressed.

Therefore, here comes the other type of study that works on causes stimulated by the environment. Major related theories consist of the under-stimulation model and the forced effort model, and the three-dimension taxonomy. To be more specific, the under-stimulation model suggests that boredom is endowed with a lack of challenging and attractive activities for students to deal with (Larson and Richards, 1991). By contrast, Hill and Perkins (1985) proposed in the forced-effort model that boredom is the consequence of numerous compulsive tasks imposed on students. Although the second type of study signifies the influence of external factors, such as tasks and activities, its focus is still limited. More fresh insights are urgently required in this line by combining people-centered and environment-based factors (Mercer-Lynn et al., 2014). This also means that future studies should concern both the internal learner factors and the external environment when conceptualizing boredom structure is attempted.

The limited focus expands to the measurement of boredom despite various measures for academic purposes, which are mainly on trait boredom, state boredom, and context-specific boredom. For example, Boredom Proneness Scale was developed to assess trait boredom (Vodanovich and Kass, 1990). Speaking of state boredom, one powerful scale is State Boredom Measure (Todman, 2013). For the context-specific boredom, the scale is Free Time Boredom Scale (Ragheb and Merydith, 2001). All of these tools for measurement can contribute to a better understanding of the nature, features, and dimensions of boredom.

### Boredom and L2 classrooms

Attention to the boredom in classrooms has been an uprising as scholars carried out researches in L2 classes by using quantitative, qualitative, and mixed approaches.

Initially, efforts are condensed in different cultural contexts such as Poland, Iran, China, and the United States. For example, in Poland, Zawodniak et al. (2017) selected 30 English major students and used students’ diaries for a period of 2 months. This study found that students experienced less boredom in pragmatic English courses. In China, Li (2021) found that students got bored when they were overwhelmingly challenged or when they received scarce instructions. In Iran, Yazdanmehr et al. (2021) tracked the boredom of an adult German learner and found that boredom could be associated with the student’s engagement in the classroom. Despite the variety of cultural contexts of the classroom, all the findings have one similarity. That is when the participants are engaged in the directional, engaging, and ability-matched activity, they are less likely to feel bored.

What is mentioned above motivates researchers to take further steps. L2 classroom is classified into two types, i.e., traditional classroom and online classroom. For the first type, scholars tried to link boredom with positive psychology. Dewaele and Li (2021) analyzed the correlation between perceived teacher enthusiasm, emotions (enjoyment and boredom), and social-behavioral learning engagement. This implies that positive and negative emotions cannot be separated. The second type is incentivized by the raging of COVID-19 which promotes online classes and causes a drastic shift in education modality. Thus, boredom in the online L2 classroom becomes a keen research topic to examine learner emotions in the new context. Derakhshan et al. (2022) attempted to find out what kinds of activities are more likely to induce boredom in online classes by using open-ended questionnaires to survey 240 English major students. Moreover, Derakhshan et al. (2021) explored causes of and solutions to boredom with 208 English major students based on online classes in Iran.

It should be celebrated that the above-mentioned studies have gathered good evidence from different L2 classrooms in various cultural contexts. Be as it may, these studies are mainly on English classrooms because English is still the most pervasive language worldwide, which creates the prerequisite for the present study to include other languages. On top of that, the present study holds that the type of L2 classroom can be more specified based on the students’ majors. For those non-foreign-language-major students in China, on many occasions, they just need to take the L2 classes regularly and finish tasks such as reading and writing. By contrast, students who major in foreign languages are required to be professional in translation due to requirements from the course and future jobs, with a sharp increase in bilingual transformation during the course and daily life. In another word, they receive rigorous training and always encounter strong disappointment because of the difficulty in translation. However, their mental well-being remains underexplored, as traditional views are solely on the mode of teaching and training methods when it comes to the cultivation of translators. In an effort to find high-efficient training modes, Zhao and Zi (2021) explored the mode of teaching written translation with online corpus platforms. For interpretation, researchers (Liu and Feng, 2019) found that if the class is output-centered, students are prone to improve their working memory and passion to engage in the class. Along with these studies, the training method also raises researchers’ interests. For example, Jiang (2021) believed that written translation, to some degree, is re-writing. After analyzing several cases, she concluded that re-composition is the nature of translation. Additionally, she suggested that teachers and students reacquaint translation and adopt strategies of recomposing during training. For interpretation, Liu et al. (2021) reviewed the previous empirical studies and proposed hypotheses about the relationship between working memory and interpretation.

These findings are meaningful in the curriculum development of translation classrooms. Nevertheless, most scholars ignore the impact of emotions on students. How to help foreign language major students confront negative emotions becomes a question to be addressed. Therefore, given that boredom is frequently experienced by learners, it is principal to investigate this aversive emotion in translation classes.

Using a sequential mixed-methods approach, the present empirical study aims to resolve these questions:

1. Is the revised scale applicable to Chinese foreign language major students in translation classrooms?
2. How significant does each boredom factor impact students in the translation classroom?
3. What are the targeted students’ perceptions of boredom in the translation classroom?

## Research design

### Participants

Selecting foreign major college students from different grades as the participants, the present study employed a stratified random sampling. As such, students participated in this research based on their willingness, fully aware of their rights to withdraw from the study at any time for any discomfort. And they were notified that their answers would be kept confidential. On top of that, the survey was administered to 517 students in China. After excluding the invalid ones that have highly similar answers among items, 483 remained, or 93.4% of the total. Everyone’s WeChat ID is different from each other. Moreover, a WeChat ID can be used only once to prevent robots or computer softwares from answering the questionnaires or any other potential disturbances. So participants were identified through their WeChat IDs.

The sample size should be 10–15 times more than the number of observed variables, according to Hair et al. (2009). In the adapted scale of the present study, there are 32 observed variables. Therefore, 483 samples should be proper. The rationale behind limiting the sample to foreign major college students was because these students were expected to have had more exposure to translation and had developed their own understanding than other students. In addition, this would eliminate participants’ weaknesses in the foreign languages, which makes the results more significant.

### Instruments

The present study collected the quantitative data *via* WeChat, a Chinese multi-purpose messaging app, together with an online questionnaire platform called Wenjuanxing. The questionnaire had two sections: (1) The demographic information section requested students’ gender, age, major, and year level. (2) The translation classroom boredom section comprised boredom items (see Appendix 1 for a full version of the questionnaire). The second section contained one revised scale based on the foreign language learning scale (Li et al., 2021). To make this scale adapted to the translation classroom in China, modifications were made by changing keywords in the original scale. The final version contains seven factors: translation classroom boredom, under-challenging task boredom, PowerPoint presentation boredom, homework boredom, teacher-dislike boredom, general learning trait boredom, over-challenging, or meaningless task boredom. In total, the measure has 32 items using a 5-point scale which includes absolutely disagree (1), disagree (2), neutral (3), agree (4), and absolutely agree (5).

Qualitative data were gathered offline by inviting 15 foreign language major students. With a semi-structured interview, two open-ended questions were asked regarding boredom factors of foreign language major students in translation classrooms.

### Procedure

The present study was operationalized in two stages: the questionnaire phase and the semi-structured interview phase.

In the first stage, to meet the purposes of this study, by distributing the valid questionnaires online, data were collected in the middle of December 2021. The questionnaire was imported into Wenjuanxing and then presented to students in the form of a WeChat link. By clicking the link, students can get access to the questionnaire. Altogether, 483 valid questionnaires were gathered by the end of December and gleaned from different colleges and universities in China. In the second stage, in the second stage, 18 participants will be selected to further engage in the present study were asked to answer the interview questions and submit the questionnaires on the same online platform.

In order to guarantee the trustworthiness of this study, all participants would be fully informed of how to fill out the questionnaires and assured that their responses and personal information would be remained confidential. They were also notified of their legitimacy to free withdrawal from the study at any time if they feel any discomfort. As the participants made no contact with the researcher, there were no conflicts of interest between the researcher and respondents. Then, the collected responses were double-checked for possible mistakes before being processed by R and MAXqda software for further statistical analysis. In the final step, the probe into the research questions was conducted based on the data.

## Results

### Descriptive statistics

The samples for quantitative analysis consist of 483 Chinese foreign language major students, whose ages ranged from 17 to 28, with the level of education from freshman to third-year graduate. Including both genders (male = 75, female = 408), they mainly came from Henan Province (340/70.39%) and other provinces and municipalities (Beijing, Shanghai, Tianjin, Chongqing, Anhui, Fujian, Gansu, Guangdong, Guangxi, Hainan, Hebei, Hubei, Jilin, Jiangsu, Jiangxi, Liaoning, Shandong, Shanxi, Shaanxi, Sichuan, Hong Kong, Xinjiang, Yunnan, Hunan, and Guangzhou; 143/29.60%). The source comes from participants’ IP addresses which are collected by Wenjuanxing automatically. It is also worth noting that 356 students are English majors, which consists of the majority. And 281 students’ age range is from 21 to 28, occupying 58.1% of the total. Further demographic information is demonstrated in Table 1.

**TABLE 1:** Descriptive information of participants.

Background information	No.
**Gender**	
Male	75
Female	408
**Level of education**	
Freshman	27
Sophomore	63
Junior	230
Senior	113
First-year graduate	23
Second-year graduate	10
Third-year graduate	17
**Major**	
English	356
Translation	53
French	11
German	6
Japanese	18
Russian	6
Other majors related to foreign languages	33
**Age range**	
17–18	28
19–20	174
21–28	281

### Reliability and validity tests

Fornell and Larcker (1981) hold that the factor loading should be more than 0.5, the construct reliability should be greater than 0.6 and the average variance extracted (AVE) should exceed 0.5. Table 2 shows that factor loadings range from 0.472–0.919 and the construct reliability ranges from 0.674 to 0.926. Moreover, the convergence validity yields acceptable results, varying from 0.428 to 0.618. Based on these results, the next step is ready to take.

**TABLE 2:** Analysis of construct reliability and convergence validity.

Construct indicator		Sig. test of parameters				Std.	Item reliability	Construct reliability	Convergence validity
				
		Unstd.	SE	*Z*-value	*P*		SMC	CR	AVE
Translation classroom boredom	A1	1.000				0.685	0.96	0.926	0.618
	A2	1.157	0.081	14.244	***	0.793	0.96		
	A3	1.233	0.042	14.966	***	0.845	0.96		
	A4	1.265	0.043	14.909	***	0.867	0.96		
	A5	1.174	0.038	15.380	***	0.805	0.96		
	A6	1.288	0.041	15.495	***	0.883	0.96		
	A7	1.341	0.039	16.108	***	0.919	0.95		
	A8	1.303	0.040	15.701	***	0.893	0.96		
Under-challenging task boredom	B1	1.000				0.693	0.96	0.836	0.506
	B2	1.137	0.086	13.274	***	0.788	0.96		
	B3	1.155	0.087	13.244	***	0.800	0.96		
	B4	1.187	0.089	13.327	***	0.823	0.96		
	B5	1.179	0.086	13.645	***	0.817	0.96		
PowerPoint presentation boredom	C1	1.000				0.472	0.96	0.674	0.428
	C2	1.695	0.189	8.970	***	0.801	0.96		
	C3	1.723	0.194	8.878	***	0.814	0.96		
Homework boredom	D1	1.000			***	0.763	0.96	0.851	0.586
	D2	0.997	0.068	14.773	***	0.761	0.96		
	D3	1.047	0.063	16.571	***	0.799	0.96		
	D4	1.147	0.065	17.623	***	0.875	0.96		
Teacher-dislike boredom	E1	1.000			***	0.800	0.96	0.818	0.533
	E2	1.209	0.071	17.033	***	0.967	0.96		
	E3	0.997	0.073	13.636	***	0.798	0.96		
	E4	0.960	0.068	14.103	***	0.768	0.96		
General learning trait boredom	F1	1.000			***	0.744	0.96	0.851	0.540
	F2	1.019	0.070	14.620	***	0.758	0.96		
	F3	1.115	0.071	15.816	***	0.830	0.96		
	F4	0.923	0.066	14.027	***	0.687	0.96		
	F5	1.214	0.077	15.754	***	0.903	0.96		
Over-challenging or meaningless task boredom	G1	1.000			***	0.781	0.96	0.728	0.482
	G2	1.152	0.083	13.848	***	0.900	0.96		
	G3	0.958	0.079	12.078	***	0.748	0.96		

*** The value is signifcant at p < 0.001

In Table 3, there exhibits AVE, covariances among latent variables, and the arithmetic square root of AVE. The lower triangular matrix is the covariance. Values on the diagonal are the arithmetic square root of AVE. According to Bagozzi and Yi (1988), the arithmetic square root of the AVE should be greater than the absolute value of the covariance between the constructs. Therefore, as is shown in the table, the discriminatory validity can be accepted and the confirmatory factor analysis (CFA) model can be built.

**TABLE 3:** Discriminatory validity.

Latent variable	AVE	Translation classroom boredom	Under-challenging task boredom	PowerPoint presentation boredom	Homework boredom	Teacher-dislike boredom	General learning trait boredom	Over-challenging or meaningless task boredom
Translation classroom boredom	0.618	0.786						
Under-challenging task boredom	0.506	0.874	0.711					
PowerPoint presentation boredom	0.428	0.534	0.729	0.654				
Homework boredom	0.586	0.822	0.894	0.520	0.765			
Teacher-dislike boredom	0.533	0.799	0.801	0.622	0.719	0.730		
General learning trait boredom	0.540	0.758	0.732	0.331	0.759	0.572	0.734	
Over-challenging or meaningless task boredom	0.482	0.769	0.852	0.705	0.686	0.701	0.710	0.694

### First-order confirmatory factor analysis

Confirmatory factor analysis was next applied to the 32 items to examine the goodness-of-fit of the seven-factor model built based on the questionnaire. All analyses were carried out using the R language. Drawing on the experience of previous studies (Meng and Zhu, 2017; Yang and Mei, 2020), CMIN/DF, GFI, AGFI, RMSEA, SRMR, and CFI were selected to assess the model fit. The CFA results showed the fit indices were at or close to the required level, suggesting a good fit for the tested model (χ^2^/DF = 3.80; GFI = 0.918; AGFI = 0.896; RMSEA = 0.076; SRMR = 0.053; CFI = 0.872). Table 4 is the summary of the results.

**TABLE 4:** Results of goodness-of-fit indices of the model.

Model fit index	Criterion	Model fit of research model
Normed Chi-square (χ^2^/DF)	1 < χ^2^/DF < 3	3.80
GFI	>0.9	0.918
AGFI	>0.9	0.896
RMSEA	<0.08	0.076
SRMR	<0.08	0.053
CFI	>0.9	0.872

**FIGURE 1:**
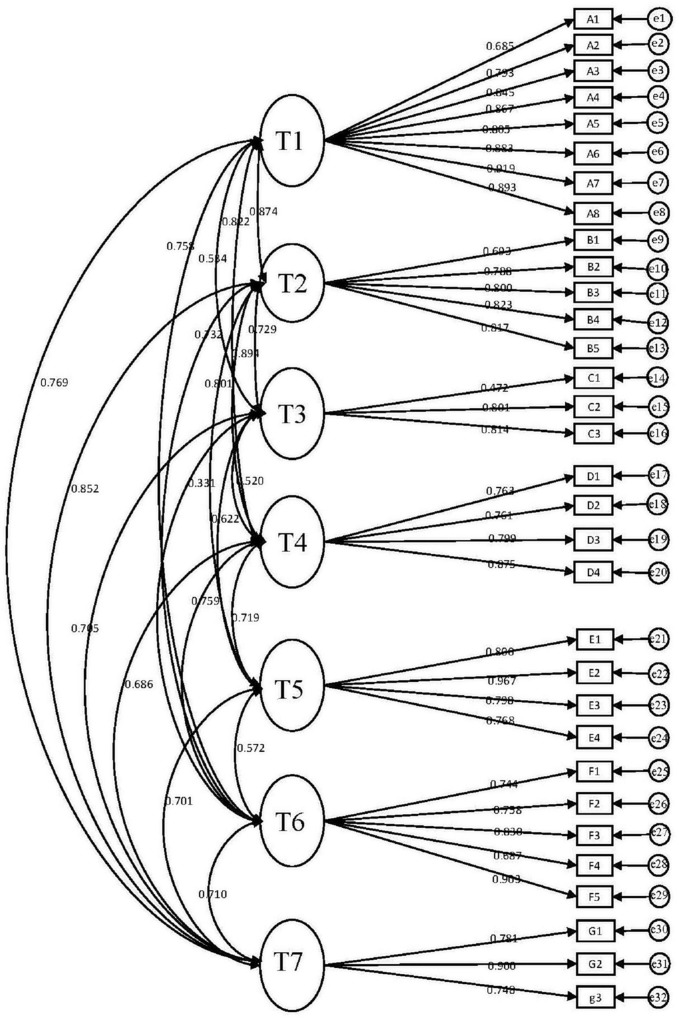
Confirmatory factor analysis model. T1, translation classroom boredom; T2, under-challenging task boredom; T3, powerpoint presentation boredom; T4, homework boredom; T5, teacher-dislike boredom; T6, general learning trait boredom; T7, over-challenging or meaningless task boredom.

Figure 1 delineates the first order of CFA to demonstrate the relationship among the observed variables and latent variables. Factor loadings and covariance were marked in the figure. Details of the name of items (A1–G1) can be seen in Appendix 1.

### Second-order confirmatory factor analysis

The current research also hypothesized and examined the second-order CFA. The second-order latent variable can be situated as an explanatory variable to an endogenous variable. By assuming the boredom in translation classes as the endogenous variable, this study conducted the analysis. Table 5 shows the goodness-of-fit. Results were acceptable.

**TABLE 5:** Results of goodness-of-fit indices of the model.

Model fit index	Criterion	Model fit of research model
Normed Chi-square (χ^2^/DF)	1 < χ^2^/DF < 3	3.97
GFI	>0.9	0.911
AGFI	>0.9	0.891
RMSEA	<0.08	0.079
SRMR	<0.08	0.060
CFI	>0.9	0.860

**FIGURE 2:**
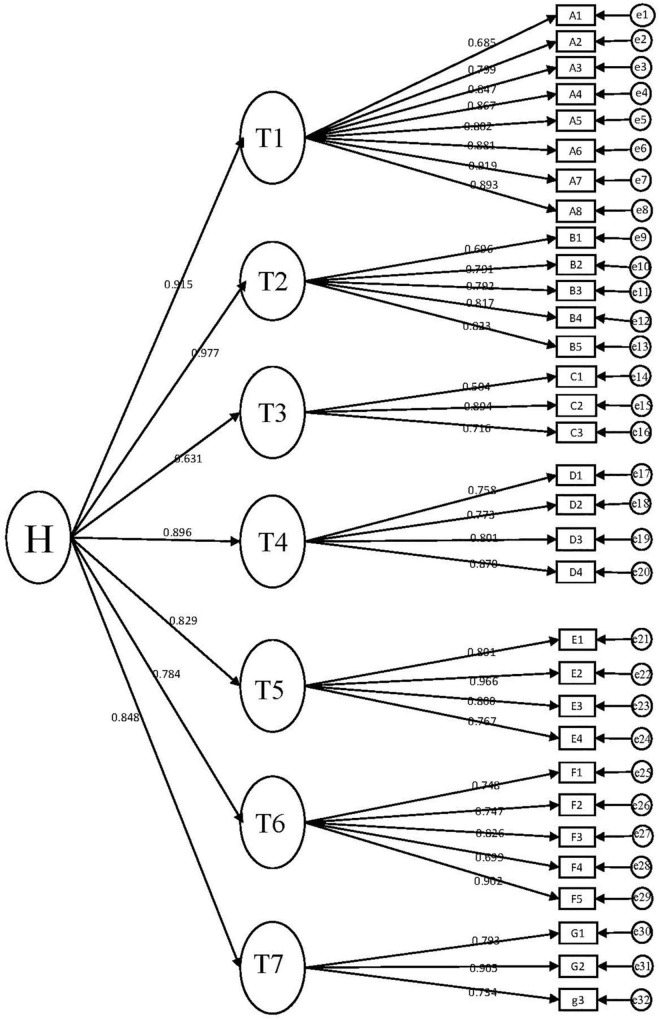
Hypothesized second-order CFA. T1, translation classroom boredom; T2, under-challenging task boredom; T3, powerpoint presentation boredom; T4, homework boredom; T5, teacher-dislike boredom; T6, general learning trait boredom; T7, over-challenging or meaningless task boredom; H, boredom in translation classes.

Figure 2 displays the results of the hypothesized second-order factorial structure for the current study. According to Figure 2, the path coefficients for boredom factors in translation classes in the hierarchical model varied among sub-constructs: foreign language classroom boredom (0.945), under-challenging task boredom (0.977), PowerPoint presentation boredom (0.631), homework boredom (0.896), teacher-dislike boredom (0.829), general learning trait boredom (0.784), over-challenging or meaningless task boredom (0.848). All the values are desirably acceptable, with details of the factor loadings of CFA and arrows drawn in Figure 2.

### Semi-structured interview

In order to elucidate perceptions of Chinese foreign language major students about boredom in translation class, this research selected 15 participants (male *N* = 6; female *N* = 9) from 7 universities through snowball sampling. All participants were sophomores and juniors of foreign language major students. The rationale of the criteria is that these students are massively exposed to translation classes because they have to prepare for high-stake exams.

The researcher carried out the interview face-to-face with all participants fully aware of the purpose of this study. It took almost half an hour to conduct the interview each time. Questions were proposed by the researcher and the assistant recorded the whole process. When sorting the interview data, the researcher standardized the text of the oral answer by removing the broken language and repeated statements on the basis of transcribing the record word by word. After double-checking the recording data, the author ensures the accuracy of the texts for the subsequent thematic analysis.

**FIGURE 3:**
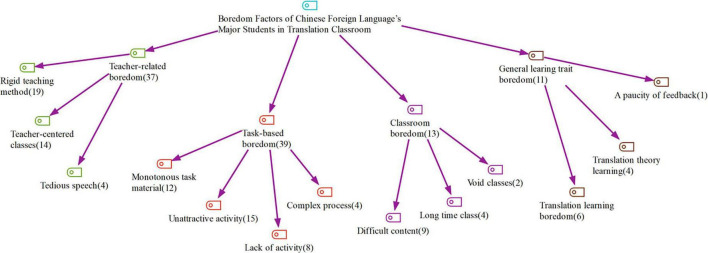
Perceptions of boredom in the translation classroom.

The thematic analysis of the relevant data ended in four general themes along with their sub-themes. The broad themes were “*teacher-related boredom*,” “*classroom boredom*,” “*task-based boredom*,” and “*general learning trait boredom*” (Figure 3).

Among these grand themes, task-based boredom was the most frequently mentioned cause of boredom in translation classrooms in China (39 references). This cause consisted of four sub-themes, namely, *complex process* (4 references), *unattractive activity* (15 references), *lack of activity* (8 references), and *monotonous task material* (12 references). Among the sub-themes, task material was the main cause of boredom for the participants who considered “*improper materials, complex content and uninteresting topics*” as the major source of boredom for Chinese foreign language major students in translation classes. The next sub-theme is “*unattractive activities*.” Participants mentioned, “*Activities are all rigid, not involving students’ thinking process, with teacher dominate the whole activity.”* Additionally, there were two less frequent sub-themes under the task-based boredom, including complex processes and lack of activities.

The second most frequently occurring theme was teacher-related boredom (37 references). They included the following sub-themes: *rigid teaching method* (19 references), *teacher-centered classes* (14 references), and *tedious speech* (4 references). Participants agreed that they were not in favor of teacher-centered classes, with the teacher “*continuing to talk and talk like a machine on contents of PPTs or textbook no matter whether students understand it or not*,” which ultimately makes students lose their interest.

Then, the theme general learning trait boredom was elucidated, with sub-themes *translation learning boredom* (2 references), *translation theory learning* (12 references), and *a paucity of feedback* (3 references). Participants reported that learning translation theory was boring with philosophical ideas that could not be applied in the translation practices. Then, a lack of feedback on the finished assignment was complained by some interviewees. Even if there were references, few participants expressed their distaste for translation learning.

The theme with the lowest mentioned frequencies was *classroom boredom* (13 references). The category was made of three sub-themes: *difficult contents* (9 references), *long-time classes* (4 references), and *void classes* (2 references). According to some participants, the contents of the translation class were extremely difficult for them because they do not have background information about it as the teacher did not give a preview. In this regard, participants pointed out, “*teachers would ignore students’ capacity to digest the information*.” Moreover, the class was always so long that students became sleepy in the end. Finally, “*void classes*” with only two references were stated by students as they felt they did not learn anything after the class.

## Discussion

### Confirmation of the revised scale

With respect to the first research question, the present study examined the modified 32-item foreign language learning scale and confirmed it as a psychometrically valid and reliable instrument for measuring the boredom of Chinese foreign language major students in the translation classroom. Referring to the results showed by CFA, it is obvious that all the factor loadings greater than 0.6 except for C1, yielding 0.472, which does not undermine the final result. What is more, the item reliability, construct reliability, and convergence reliability all reach 0.5, which supports the necessity for the subsequent step. The outcome of the model fits is satisfactory with major indicators reaching the normal standards. Therefore, all results confirm the effectiveness of the adapted questionnaire in a different context and provide the pre-requisite for the second-order CFA to determine how different boredom factors contribute to the translation classroom boredom. Given that seven constructs are correlated for further exploration, the second-order CFA was thus conducted by hypothesizing a common higher-level factor, which is the boredom in the translation classroom itself.

### Impacts of different boredom factors

In unraveling the impact of different boredom factors, to answer the second question, the findings show that all the factor loadings are higher than 0.6, which indicates that the seven constructs account significantly for the translation classroom boredom, with the under-challenging task boredom ranking the highest and PowerPoint presentation boredom the lowest.

The under-challenging task construct consists of five aspects. Among these, the one with the highest factor loading is “the translation-related exercise lasts too long.” This is tentatively indicative of the fact that the experience of boredom in the translation classroom is likely to increase as students continuously work on the same and relatively easy tasks. This result also echoes the finding of Pawlak et al. (2020) who finds that students are thirsty for challenging and satisfying tasks during language learning and the lack of proper tasks is responsible for boredom in the language class. It can be induced that the task in the translation classroom is a main source of boredom, which poses challenges to teachers who should design properer tasks based on students’ feedback.

What mentions above is also in line with the finding of over-challenging or meaningless task whose factor loading is as high as 0.848. This means that the in-class task indeed plays a vital part in the translation classroom, which may result from the fact the translation classroom concentrates more on nurturing practical skills to cope with materials. It is also worth noting that the most influential item is G2 which reads “When the translation teacher seems unmotivated to teach, I lose my motivation to listen to him/her as well.” As to this point, it resonates with Dewaele and Li (2021) that teachers’ enthusiasm, interpreted by learners, can affect students’ engagement in classes. It is therefore reasonable to say that teachers should love serving as a bridge to gap the difference between two languages before they are to light the passion of their students. If the teacher is considered to be reliable and well-prepared, students tend to devote more efforts and express their opinions (Zepke and Leach, 2010). This is also true conversely, which is also why teacher-dislike boredom gains highly as the item that writes “the translation teacher is an uninteresting, so the translation class is dull” scores 0.966. On the one hand, educators and teachers are encouraged to accept this finding and admit that there is still a large room for them to enhance their teaching and communication skills. On the other hand, it should be pointed out that students will blame their teachers innerly even if they remain silent about classes’ boredom. Therefore, how to promote students talking about their mental states will remain a topic for future studies.

Plus, more concerns should be related to translation classroom boredom which presents students’ overall feeling of the translation classroom when they are in it. Unfortunately, its factor loading ranks the second among the seven constructs. The finding manifests that the translation classroom leaves students with a deep impression of being boring. It implies that students unconsciously believe the translation classroom is boring in nature. The impact of this cognition is detrimental to students’ learning of translation in the long term as the classroom is the major place to study essential knowledge points. If they are discouraged in the first place, their efficiency will decrease accordingly. So it is urgent to refine the classroom before numerous young talents of translation are ruined by boredom.

There is no doubt that homework boredom is also a contributing factor. After all, home is not a place to work but for refreshment. Given a comfortable environment, learners are surely reluctant to finish their homework. But slightly contrary to the finding of Zawodniak et al. (2021) who concludes that learning leads to boredom, the present study finds that learning is less likely to cause boredom compared with other sources with the learning trait boredom being the second lowest. Together with other findings, this implies that students like to learn translation but teachers and improper tasks fail their expectations to some extent.

To the researcher’s great surprise, PowerPoint presentation boredom is the lowest frequently experienced. Arguably, it should be on the top of the list as the situation is always like this in China: with total indulgence in their own worlds, teachers read PowerPoint as if they were robots even though students are absent-minded, lost or confused. This counterfactual finding is probably because PowerPoint is not considered as the major source. Instead, students attribute their boredom mainly to teachers. This can explain why teacher-dislike boredom is relatively higher among students.

### Perceptions of boredom in translation classes

The third research question is designed to provide insights for underpinning the empirical results. The finding first shows that students attribute boredom to inappropriate and unexpected tasks. This result is consistent with the empirical analysis, showing that task-based boredom is the most influential in the translation classroom. It is aligned with Peacock (1997), who found that good materials could impact positively the EFL learners, which could increase the learners’ engagement. Therefore, teachers are encouraged to prepare original materials and tasks on the latest topics for students with the help of the internet and visualization tools. Another finding again emphasizes the leading role that teachers play in the translation classroom. Teacher-related boredom is raised as claims, including “teacher-centered classes,” “rigid teaching method,” and “tedious speech” are against translation teachers. This, in turn, explicates the teacher-dislike boredom in the empirical section. In contrast to the student-centered classroom, the teacher-dominated class can discourage students to respond to stimuli (Serin, 2018). Students are suggested to express themselves in the classroom as participants comment on teachers’ tedious speech. One interviewee in this study reported that there was no chance to show the translation result in the class nor sufficient feedback for her to improve. As there was nothing to learn, her boredom with the translation class thus came into being. Similarly, other scholars (Chen et al., 2022) also find that teachers gave scant chance for students to express themselves in class, which escalated students’ level of boredom.

In addition, general learning trait boredom and classroom boredom were found but they are not as frequently mentioned as teacher-related boredom, especially the latter. This is not aligned with the empirical study. The reason could be that the number of participants is limited and the influence of classroom boredom is thereby not manifested. It is suggested that more efforts should be made to study this domain-related boredom. Moreover, combined with the empirical results, the qualitative data elaborate on the learning trait boredom in terms of the learning content. In this dimension, it should be pointed out that students are unwilling to learn translation theories that they believe are redundant. Many participants mentioned that many translation theories are too philosophical to understand, let alone master. One of the interviewees directly remarked, “Theories are useless. Diving into the sea of translation exercises and practices is much more effective.” Hence, this finding gives strong evidence for educators to make the translation theories more interesting, at least easier to understand.

## Conclusion

By building a structural equation model and conducting a thematic analysis, the perception and factors of boredom of Chinese foreign language major students in translation classes were investigated. The statistical results first confirm the credibility and reliability of the adapted questionnaire and then demonstrate each construct’s contribution to the boredom during the translation learning. Corroborated by the thematic analysis that gives a picture of the current situation, it is fair to conclude that task-based boredom and teacher-related boredom are the most frequently experienced by foreign language major students in the translation class.

The findings of the present study have left meaningful implications. Firstly, the present study validated the adapted scale to measure the boredom of foreign language major students in the translation class for future studies to refer to. Secondly, the present study suggests more engaging tasks with the proper level of difficulty should be introduced to the translation classroom. The translation texts for practice should be on the latest topics. Teachers are also expected to improve their teaching styles, refine their instructional skills and give more opportunities to students to shine in the class. Moreover, the present study can help teachers to identify the boredom source so that they can reduce the boredom-inducing tasks and activities in the translation classroom. Foreign language major students can thus be benefited and be more prepared for their future careers.

Having said that, this study reasonably suffers from certain limitations. First of all, the present study was carried out in the translation classroom context including several different majors. Thus, the findings of this study cannot be generalized to other contexts. Secondly, the effects of other factors, such as gender, age, and major, were not considered nor specifically controlled. To make findings more accurate, further studies are advised to do so.

## Data availability statement

The original contributions presented in this study are included in the article/supplementary material, further inquiries can be directed to the corresponding author.

## Author contributions

TZ contributed to the design and implementation of the research, analysis of the results, and writing of the manuscript.

## Appendix

**Appendix 1:** The revised questionnaire.

Item code	Items
A1	The translation class bores me.
A2	I start yawning in translation class because I’m so bored.
A3	My mind begins to wander in the translation class.
A4	I am only physically in the class room, while my mind is wandering outside the translation class.
A5	It is difficult for me to concentrate in the translation class.
A6	Time is dragging on in translation class.
A7	I get restless and can’t wait for the translation class.
A8	I always think about what else I might be doing to kill the time rather than sitting in this translation class.
B1	I believe an analysis of long text in translation is really dreary.
B2	It is really boring to repeat the text after the listening to the audio.
B3	So many similar types of exercise make me lose interest.
B4	So much practice on a same (translation-related) subject matter makes me restless.
B5	The (translation-related) exercise or a subject matter lasts too long, and I feel bored.
C1	It would have more interested if other multimedia resources were utilized in class rather than PPT slides loaded with text.
C2	PPT slides filled up with solely script but without interactions make me bored.
C3	Reading from script in the PPT slides bores me.
D1	Just thinking of my translation homework makes me feel bored.
D2	I get bored of too much translation homework.
D3	Translation homework is over-challenging and I don’t want to do it.
D4	Doing translation homework is a dull activity.
E1	I am not interested in translation class, because the translation teacher isn’t likable.
E2	The translation teacher is an uninteresting, so the translation class is dull.
E3	I really dislike the translation teacher spending so much.
E4	I feel agitated because the translation teacher spends too much time saying things that are irrelevant to the teaching material.
F1	I’m always bored when I study.
F2	I’m somebody who is not interested in study.
F3	Not only learning translation, studying is dull in general.
F4	Other subjects are similarly boring and dull like translation.
F5	I’m forced to learn all the subjects including translation.
G1	I don’t care about teaching and learning activities that the translation teacher does not value.
G2	When the translation teacher seems unmotivated to teach, I lose my motivation to listen to him/her as well.
G3	If I cannot understand classmates’ presentations, I become really bored.

## Appendix 2

Interview questions.

1. What are the factors of boredom while you are having translation classes?
2. Do you have any further comments on the translation classes?
